# Importance of Neutrophil Gelatinase-Associated Lipocalin in Differential Diagnosis of Acute and Chronic Renal Failure

**DOI:** 10.5812/ircmj.14133

**Published:** 2014-08-05

**Authors:** Seda Ozkan, Polat Durukan, Cemil Kavalci, Ali Duman, Mustafa Burak Sayhan, Omer Salt, Afsin Ipekci

**Affiliations:** 1 Department of Emergency Medicine, Diskapi Training and Research Hospital, Ankara, Turkey; 2 Department of Emergency Medicine, Faculty of Medicine, Erciyes University, Kayseri, Turkey; 3 Department of Emergency Medicine, Faculty of Medicine, Baskent University, Ankara, Turkey; 4 Department of Emergency Medicine, Isparta State Hospital, Isparta, Turkey; 5 Department of Emergency Medicine, Faculty of Medicine, Trakya University, Edirne, Turkey; 6 Emergency Department, Yozgat State Hospital, Yozgat, Turkey

**Keywords:** Renal Failure, Chronic, Acute, NGAL Protein

## Abstract

**Background:**

Neutrophil Gelatinase-associated Lipocalin (NGAL) protein is easily detected in the blood and urine soon after acute renal injury. NGAL gains features of an early, sensitive and noninvasive biomarker for acute renal injury. Recent evidences suggest that its expression is also increased in CRF reflecting the severity of disease.

**Objectives:**

In the present study, we aimed to investigate whether blood NGAL level plays a role in the differential diagnosis of acute and chronic renal failure.

**Patients and Methods:**

This was a prospective case-control study. Fifty patients presented to emergency department with acute renal failure (ARF), 30 with chronic renal failure (CRF) and 20 healthy individuals as control group were included in this study. Blood pH, HCO3^-^, BUN, creatinine and potassium values were evaluated in all patients. Blood NGAL values were evaluated in all groups. BUN, serum creatinine and NGAL values were statistically compared between patients and controls.

**Results:**

Median NGAL levels in patients was 304.50 (29), and 60 (0) in control, which was statistically significant between the two groups (Z = -6.477, P < 0.001). The median NGAL values were 261.50 ± 291 in ARF group and 428.50 ± 294 in CRF group. There was a significant difference in NGAL level between ARF and CRF groups (Z = -2.52, P = 0.012). Median BUN values were 153.46 ± 82.47 in ARF group and 169.40 ± 93.94 in CRF group. There was no significant difference in BUN value between ARF and CRF groups (P > 0.05). Median creatinine values were 2.84 ± 2.95 in ARF group and 4.78 ± 4.32 in CRF group. In serum creatinine values, a significant difference was found between ARF and CRF groups (P < 0.05).

**Conclusions:**

Serum NGAL levels of ARF and CRF patients were significantly higher than healthy individuals. In addition, NGAL values of patients with CRF were significantly higher than those of ARF. Serum NGAL values can be used to detect renal injury and differentiate ARF and CRF.

## 1. Background

Acute renal failure (ARF) refers to a condition, in which a reduction occurs in glomerular filtration rate (GFR) causing accumulation of blood urea nitrogen (BUN), creatinine and other uremic toxins in the body. In ARF, GFR reduction is relatively faster and develops within days or weeks compared to chronic renal failure, in which GFR reduces within months or years (1). Chronic renal failure (CRF) is defined as chronic and progressive impairment in kidney function as a result of reduced GFR, including fluid-solute homeostasis and metabolic-endocrine functions. End-stage renal disease (ESRD) is considered, when GFR decreases to 5-10 mL/min; these patients need renal replacement therapies such as dialysis and renal transplantation (2). ARF is rarely a community-acquired disease as it usually develops in hospitalized patients. ARF may complicate the existing disease in 5% of hospitalized patients; among these, dialysis may be needed in 0.05% (3-5). This rate may be substantially high up to 20% in patients with severe disease (5, 6). Mortality rate varies from 7% to 80% depending on the severity of renal failure (7, 8). ARF diagnose is based on clinical findings (history and physical examination); however, biochemical confirmation is needed. Initial examination of a patient with ARF should include the diagnosis of renal failure and evaluations to determine the etiology and severity of ARF. However, evaluations at early stage (plasma bicarbonate, complete blood count, urine analysis, urine biochemistry, renal ultrasonography) are focused on the confirmation of diagnosis and life-threatening disorders. Complex tests (specific immunological assays, radioisotope scintigraphy and renal biopsy) aimed to establish specific diagnosis are less needed (9). BUN and serum creatinine levels are elevated in renal failure. There is a need for further tests to distinguish acute and chronic renal failure. Presence of previous increases in BUN and creatinine, abnormal urine analysis and creatinine known to be stable but elevated are supportive of chronic renal failure. Anemia, metabolic acidosis, hyperphosphatemia, hypocalcaemia and hyperkalemia are seen both in acute and renal failure. GFR measurement is the best method to determine the degree of renal function. Renal function should not be assessed by considering serum creatinine concentration. In ultrasonography (USG) evaluation, presence of bilaterally small (< 9 cm), echogenic kidneys suggests the diagnosis of CRF (10). Neutrophil Gelatinase-associated Lipocalin (NGAL) is a glycoprotein with a molecular weight of 25-kDA found in neutrophil granules and expressed at low concentrations in normal kidney, trachea, lungs, stomach, and colon (11, 12). NGAL is expressed and secreted from renal tubular cells, hepatocytes and immune cells in several pathological conditions (13). Its expression is also induced in injured epithelia, including lung, colon, and especially kidney (12). NGAL, a new member of lipocalin, is excreted by urine and can be readily detected, because it has a low molecular weight as well as resistance to fragmentation. NGAL accumulates in human renal cortical tubules, blood and urine after nephrotoxic and ischemic injuries (14).

## 2. Objectives

 NGAL protein is easily detected in the blood and urine soon after acute renal injury. NGAL gains features of an early, sensitive and noninvasive biomarker for acute renal injury (15). Recent evidences suggest that its expression is also increased in CRF reflecting the severity of disease (16). In our study, we aimed to investigate whether serum NGAL plays a role in the differential diagnosis of acute and chronic renal failure.

## 3. Patients and Methods

This study was conducted in 2011 at Erciyes University Faculty of Medicine Department of Emergency Medicine and Trakya University Faculty of Medicine Department of Emergency Medicine. The study was performed in a tertiary university hospital. The study was approved by the Ethics Committee (09/135) of Erciyes University, Medical Faculty, Kayseri, Turkey. Written informed consent was obtained from all patients enrolled in the study. This was a prospective case-control study. Fifty patients presented to emergency department with ARF, 30 with CRF and 20 healthy subjects as control group were included in this study. Eighty consecutive patients fulfilling the inclusion criteria and willing to participate in the study were included in the study. Twelve patients not meeting the inclusion criteria were excluded from the study.

Inclusion criteria were:

ARF Group:

1. Patients older than 18 years
2. Patients without known BUN or creatinine rise
3. Patients without known CRF
4. Patients without CRF findings in renal USG
5. Patients describing sudden decrease in urine amount or anuria

CRF Group:

1. Patients older than 18 years
2. Patient with known BUN or creatinine rise
3. Patients previously diagnosed as CRF
4. Patients with CRF findings in renal USG

Exclusion criteria were:

1. Patients younger than 18 years
2. Patients with normal levels of BUN or creatinine and without CRF

The study was designed in 95% confidence interval and P < 0.05 was considered as significant. To differentiate prerenal and renal causes of ARF, number of patients in ARF group was higher. Data of uremic patients meeting the inclusion criteria were recorded in a form previously prepared. Diabetes mellitus, hypertension and CRF were asked in patients’ history. Nausea and vomiting were recorded. In physical examination, presence of crackles and pretibial edema were assessed. Blood samples were obtained to measure pH, HCO3^-^, BUN, creatinine, potassium and NGAL at presentation to emergency department. After taking laboratory results, ARF and CRF groups were determined. Sampling was terminated when we reached the number of 50 consecutive ARF patients and 30 CRF patients. Serum NGAL values were determined by a bedside kit. The kit was calibrated by its own cartridge. Serum NGAL levels were measured on admission using a quantitative fluorescence immunoassay (Triage-NGAL Meter, Biosite Diagnostics, Ins. San Diego, California). The sensitivity of the quantitative fluorescence immunoassay assessment was less than 10 pg/mL. All patients underwent renal USG evaluation. Renal size smaller than 9 cm and increased echogenicity in renal parenchyma were interpreted in favor of CRF in renal ultrasonography. If these findings were not present, it was then interpreted as ARF. Blood NGAL values were evaluated in all groups. BUN, serum creatinine and NGAL values were compared between the control and patients groups. Statistical analysis was performed using SPSS version 18.00. According to the results of the power analysis, at α α = 0.05, β = 0.05 (power = 95%) each group should have contained at least 18 patients. Normality of data distribution was tested with Kolmogorov-Smirnov test. X^2^ test was used to compare categorical variables. Student T test was used to compare continuous variable between the groups, when distribution of data was normal. Mann-Whitney U test and Kruskal-Wallis test were used to compare continuous variable between the groups, when distribution of data was abnormal. P < 0.05 was considered as statistically significant. Power of the study was determined as 0.25.

## 4. Results

Eighty patients were included in this study. Fifty patients (62.5%) were found to have ARF, while 30 (37.5%) patients had CRF. Totally, there were 39 (48.8%) male and 41 (51.3%) female in patient groups. The number of both genders in the groups was shown in Table 1. There was no significant difference in gender (P > 0.05) (Table 1).

**Table 1.** Demographic Characteristics, Symptoms and Signs of Participants^a,b,c^

	ARF	CRF	Control
**Age, y**	67.64 ± 13.03	65.53 ± 16.38	56.3 ± 9.79
**Gender**			
Female	26 (51)	15 (9)	10 (50)
Male	24 (49)	15 (31)	10 (50)
**History**			
Hypertension	27 (56.3)	21 (43.8)	0 (0)
DM	14 (66.7)	7 (33.3)	0 (0)
CRF	6 (20)	24 (80)	0 (0)
**Symptoms**			
Nausea	16 (61.5)	10 (38.5)	0 (0)
Vomiting	16 (72.7)	6 (27.3)	0 (0)
**Signs**			
Crackle	11 (61.1)	7 (38.9)	0 (0)
Pretibial edema	15 (75)	5 (25)	0 (0)

^a^For these parameters, there was no statistical difference between the groups (P > 0.05).

^b^Abbreviations: DM, diabetes mellitus; CRF, chronic renal failure.

^c^Data are presented as No. (%) or Mean ± SD.

The mean age was 64.13 ± 14.14; it was 67.64 ± 13.03 in patients with ARF and 65.53 ± 16.38 in those with CRF (Table 1). Nausea was found in 26 (32.5%) patients, while 54 (67.5%) patients had no nausea. Among patients with nausea, ARF and CRF were diagnosed in 16 (61.5%) and 10 (38.5%) patients, respectively (Table 1). Among those without nausea, ARF and CRF were diagnosed in 34 (63.0%) and 20 (37.0%) patients, respectively. In total, 22 (27.5%) patients had vomiting, while 58 (72.5%) patients did not. Among these, ARF and CRF were diagnosed in 16 (72.7%) and 6 (27.3%) patients, respectively (Table 1). Among those without vomiting, ARF and CRF were diagnosed in 34 (58.6%) and 24 (41.4%) patients, respectively. Hypertension was found in 48 (60.0%) patients, while 32 (40.0%) patients had no hypertension. Among these, ARF and CRF were diagnosed in 27 (56.3%) and 21 (43.8%) patients, respectively (Table 1). Among those without hypertension, ARF and CRF were diagnosed in 23 (71.9%) and 9 (28.1%) patients, respectively. Diabetes mellitus (DM) was found in 21 (26.2%) patients, while 59 (73.8%) patients had no DM. Among those with DM, ARF and CRF were diagnosed in 14 (66.7%) and 7 (33.3%) patients, respectively (Table 1). Among those without DM, ARF and CRF were diagnosed in 36 (61.0%) and 23 (39.0%) patients, respectively. In their history, 30 (62.5%) patients had a CRF diagnosis, whereas 50 (37.5%) patients did not. There were six patients with CRF history in ARF group and 24 patients in CRF group (Table 1). In physical examination, crackle was found in 18 (22.5%) patients. Of patients with crackle, ARF and CRF were diagnosed in 11 (61.1%) and 7 (38.9%) patients, respectively (Table 1). In physical examination of those without crackle, ARF and CRF were diagnosed in 39 (62.9%) and 23 (37.1%) patients, respectively. In physical examination, pretibial edema was found in 20 (22.0%) patients. Of these, ARF and CRF were diagnosed in 15 (75.0%) and 5 (25.0%) patients, respectively (Table 1). Of those without pretibial edema, ARF and CRF were diagnosed in 35 (58.3%) and 25 (41.7%) patients, respectively. Renal USG results were shown in Table 2.

**Table 2.** Renal Sonography Findings in Groups 1 and 2^a,b^

Renal USG	ARF	CRF
**Normal**	33 (66)	-
**Smaller size**	-	17 (56.6)
**Increased parenchyma echogenicity**	-	27 (90)
**Hydronephrosis**	9 (18)	-
**Stone**	8 (16)	2 (6.7)

^a^Abbreviation: USG, ultrasonography; ARF, acute renal failure; CFR, chronic renal failure.

^b^data are presented as No. (%).

The median BUN value was 153.46 ± 82.47 in ARF group and 169.40 ± 93.94 in CRF group. There was no significant difference in BUN values between ARF and CRF groups (P > 0.05; Table 3). The median creatinine value was 2.84 ± 2.95 in ARF group and 4.78 ± 4.32 in CRF group. There was a significant difference in creatinine value between ARF and CRF groups (P < 0.05; Table 3). Biochemical results and blood gas analysis were shown in Table 3.

**Table 3.** Blood Gas Analysis and Biochemical Values ^a^

	ARF	CRF	P Value
**pH**	7.35 ± 0.73^b^	7.28 ± 0.91	t = 3.95, P = 0.000^c^
**pO2**	90.0 (20.03)^d^	89.0 (21.15)	z = -0.22, P = 0.822^e^
**pCO2**	35.8 (13.35)	36.5 (15.25)	z = -0.40, P = 0.968^e^
**HCO3**	18.17 ± 5.39	16.87 ± 4.38	t = 1.11, P = 0.268^c^
**K**	4.77 ± 0.95	5.14 ± 1.14	t = -1.54, P = 0.126^c^
**BUN**	153.46 ± 82.47	169.40 ± 93.94	t = -0.79, P = 0.430^c^
**Creatinine**	2.84 (2.95)	4.78 (4.32)	z = -3.03, P = 0.001^e^
**NGAL**	261.50 (291)	428.50 (294)	z = -2.52, P = 0.012^e^

^a^Abbreviations: AFR; acute renal failure, BUN; blood urea nitrogen, CFR; chronic renal failure, NGAL; neutrophil gelatinase-associated lipocalin.

^b^Mean ± SD.

^c^Median (IQR).

^d^Student T test.

^e^Mann-Whitney U test.

BUN or serum creatinine values were high in 44 (55.0%) patients. In ARF group, normal BUN or creatinine values were detected in 28 (56.0%) patients, while elevated values were detected in 22 (44%) patients. In CRF group, normal BUN or creatinine values were detected in eight (26.7%) patients, while elevated values were detected in 22 (73.3%) patients. Median NGAL levels were 304.50 (29) and 60 (0) in patient and control groups, respectively. There was a statistically significant difference between the two groups (Z = -6.477, P < 0.001). There was a significant difference in NGAL values between ARF and CRF groups (P < 0.05). The median NGAL values were 261.50 (291) in ARF group and 428.50 (± 294) in CRF group. There was a significant difference in NGAL values between ARF and CRF groups (P < 0.05, Table 3). NGAL value was elevated in 67 (83.8%) patients, while it had normal levels in 13 (16.3%) patients. In ARF group, NGAL values were elevated in 38 (76.0%) patients, while normal values were detected in 12 (24%) patients. In CRF group, NGAL values were elevated in 29 (96.7%) patients, while normal value was detected in one (3.3%) patient. Of patients with normal NGAL value, 12 (92.3%) and one (7.7%) patients were diagnosed as ARF and CRF, respectively. Of those with elevated NGAL values, 38 (56.7%) and 29 (43.3%) patients were diagnosed as ARF and CRF, respectively. Of patients with ARF, 28 (56%) were due to prerenal, seven (14%) renal and 15 (30%) postrenal causes. There was no significant difference in NGAL values between ARF subgroups (P > 0.05; Table 4).

**Table 4.** Neutrophil Gelatinase-Associated Lipocalin Values of Acute Renal Failure Subgroups^a^

	NGAL (median IQR)	X^2^	P Value^b^
**Prerenal**	272 (242)		
**Renal**	134 (96)	5.71	0.58
**Postrenal**	328 (310)		

^a^Abbreviation: NGAL, Neutrophil Gelatinase-Associated Lipocalin; IQR, Interquartile range.

^b^Kruskal-Wallis test.

## 5. Discussion

Acute renal failure (ARF) refers to a condition, in which a reduction occurs in GFR; this reduction causes accumulation of blood urea nitrogen (BUN), creatinine and other uremic toxins in the body. In ARF, GFR reduction develops within days or weeks compared to chronic renal failure, which occurs within months or even years (1). As GFR reduction may occur in individuals without preceding renal injuries, it may present acute exacerbations in individuals with known chronic renal diseases. Varying amounts of urine output are seen in ARF (3-5). Long-term and severe ischemia due to decreased renal blood flow can cause acute tubular necrosis. Therefore, restoring renal blood flow as soon as possible would limit ischemic duration at kidneys and preventing parenchyma injury. In prerenal ARF, recovery in renal functions starts within 24-48 hours after restoration of renal blood flow (1). As CRF is reflected by progressively reducing glomerular filtration values, it is defined as progressive and irreversible loss of renal function due to nephron loss over years (2). Diabetes mellitus (39%), hypertension (26%), primary glomerulonephritis (11%), hereditary, congenital or cystic diseases of kidneys (4%), interstitial nephritis/pyelonephritis (4%), secondary glomerulonephritis/vasculitis (2%), renal artery stenosis (2%), malignancy (2%), nephrolithiasis/obstructive nephropathy (1%) and AIDS nephropathy (1%) are main causes of CRF (17). The most common cause of CRF was glomerulonephritis in the past, while diabetic and hypertensive nephropathies are currently common etiologies. The reason for this shift in etiology is probably due to effective management and prevention of glomerulonephritis as well as decreased mortality, particularly, in patients with diabetes and hypertension. In general, increased life-span and decreased early cardiovascular mortality lead to an increase in the mean age of patients with CRF. Hypertension is the most common cause of CRF among elderly population (18). In a study from Turkey, diabetes mellitus and hypertension were found as primary causes of hypertension (19). In our study, hypertension was detected in 54% of patients with ARF, while it was detected in 70% of those with CRF. Moreover, DM was found in 28% of patients with ARF, while it was detected in 23.3% of those with CRF. In our study, hypertension rate was higher than that of literature for the development of ARF and CRF, while data regarding DM was consistent with the literature. Higher hypertension rates may be attributed to higher prevalence of hypertension in our region and lifestyle. However, it is apparent that controlling hypertension and DM is quite important. Clinical symptoms and signs are closely correlated with the degree of renal failure and development rate. Patients may be asymptomatic until GFR reduction to below 35-50 mL/min. Initial symptoms usually include nocturia and fatigue secondary to anemia. When glomerular filtration rate decreases to 20-25 mL/min, uremic symptoms become apparent. When it decreases to 5-10 mL/min, end-stage renal failure becomes a concern and patients usually needs renal replacement therapies such as dialysis and renal transplantation (2). Patients generally have fatigue, weakness and exhaustion due to CRF. Gastrointestinal symptoms such as anorexia, nausea, vomiting, metallic taste in mouth and hiccup are frequently seen (11). In our study, nausea was found in 32% and vomiting in 32% of patients with ARF, while these values were 33.3% and 20% in patients with CRF, respectively. This indicates that the most frequent complaints were nausea and vomiting in accordance with the literature. Therefore, all patients with nausea and vomiting should be evaluated for renal functions. In physical examination, patient seems ill; hypertension is common and yellowish skin color is striking. There is a fish odor in exhalation breathing due to uremia. Cardiopulmonary findings including crackles, cardiomegaly, edema and pericardial friction rub. Altered mental status may be seen as well (11). In our study, of patients with ARF, crackle was found in 22% as pulmonary auscultation finding and pretibial edema in 30%. Crackle and pretibial edema were detected in 23.3% and 16.7% of patients with CRF. These findings suggest that patients should be evaluated for renal dysfunction in the presence of crackle and pretibial edema in physical examination. Presence of bilateral, small (< 9 cm), echogenic kidneys in ultrasound evaluation suggests CRF. In CRF, normal or large kidneys are seen in polycystic renal diseases, diabetic nephropathy, HIV-related nephropathy, multiple myeloma, amyloidosis and obstructive uropathy (10, 11). Renal sizes and parenchyma were normal in all of ARF patients. Nevertheless, renal stone was detected in 16% and hydronephrosis in 18% of ARF patients. In the CRF group, renal USG results of all patients had abnormal findings. This indicates that renal USG may be helpful to differentiate ARF and CRF. In addition, it may be useful to detect causes of ARF and CRF. Consequently, renal USG is a rapid, easy and noninvasive method in cases, in which renal dysfunction was detected. Elevated BUN or serum creatinine levels are found in renal failure. However, further tests are needed to distinguish acute and chronic renal failure. Presence of previous increases in BUN or creatinine, abnormal urine analysis and known to be stable but elevated creatinine are supportive for chronic renal failure. Anemia, metabolic acidosis, hyperphosphatemia, hypocalcaemia and hyperkalemia are seen both in acute and renal failures (11). The most commonly used laboratory parameter is creatinine in clinical practice. Although creatinine is widely used in clinical practice, it has some disadvantages. Creatinine is affected by several factors such as age or sex and is unable to reflect acute renal injury as early as needed (20). Firstly, renal function should be impaired more than 50% for elevation in serum creatinine levels. Similarly, serum creatinine does not reflect renal function accurately until they reach to a stable condition and this stable condition of renal function needs a few days (21). Serum creatinine value is an insufficient indicator for acute renal injury (22). In our study, serum BUN or creatinine levels were normal in 56% and 26.7% of patients diagnosed as ARF and CRF, respectively. BUN and serum creatinine values may not be sufficient as markers to diagnose renal failure and differentiate ARF and CRF. Therefore, a new marker is needed for early diagnosis of renal failure and differential diagnosis of ARF and CRF.

Some alternative biomarkers such as Neutrophil gelatinase-associated lipocalin (NGAL), urinary cysteine-rich protein 61 (Cyr 61), human kidney injury molecule 1 (hKIM-1), Urinary interleukins/adhesion molecules (IL-6, IL-8, IL-18), glomerular filtration markers (Proatrial natriuretic peptide (1-98) and cystatin C), urinary glutathione-S-transferase (GST), Spermidine/spermine N-acetyl transferase (SSAT) and Actin have been developed (23). It is a small molecular size (25 kDa) lipocalin iron-carrying protein released from tubular epithelial cells upon injury. Damage markers are potentially better than functional markers. NGAL is a marker of active tubular pathology (24, 25). Serum and urinary NGAL are arguably the most promising emerging biomarkers for detection of ARF (25). The reasons for NGAL increase in ARF are acute tubular injury and NGAL secretion from neutrophils, macrophages and other immune cells as acute phase reactant (12). There is a growing literature suggesting that NGAL is also a marker of kidney disease and severity in CRF (24). It was shown in the literature that tubulointerstitial injury almost in all forms of CRF and serum NGAL increase is its result (25). In a Western Blot study conducted on patients with ARF in intensive care units, it was found that NGAL levels were elevated 10 fold in plasma and 100 fold in urine secondary to sepsis, ischemia and nephrotoxin. Both urine and plasma levels were correlated to serum creatinine levels (13). In a prospective study, acute renal injury developed in 28% of children who underwent cardiopulmonary bypass, where serum creatinine levels were elevated 1-3 days after the operation. However, NGAL levels were elevated 10 fold and detected in urine and plasma within 2-6 hours after the operation. It was concluded that both urine and plasma levels of NGAL are strong and independent markers of acute renal injury (26). In this study, serum NGAL levels in patients with ARF and CRF were significantly higher than healthy individuals. NGAL value was elevated in 76% and normal in 24% of patients in ARF group, while it was elevated in 96.7% and normal in 3.3% of patients in CRF group. It was also detected that NGAL values were higher in more patients in CRF group than ARF group. Meanwhile, NGAL values in CRF group were significantly higher than those of ARF group. Morbidity and mortality could be prevented by early diagnosis and treatment in renal dysfunction. It is not possible to detect elevated BUN or creatinine values at early stage. However, NGAL level is an important parameter could be used as an early marker in patients developing renal dysfunction when BUN or creatinine levels are not increased yet. Serum NGAL levels of ARF and CRF patients were significantly higher than healthy individuals. Furthermore, NGAL values of CRF patients were significantly higher than those of ARF patients. As a result, we concluded that serum NGAL level can be considered to detect renal injury and differentiate ARF and CRF.

### 5.1. Limitation

Small number of sample size may be the weak point of study.
